# Enteric methane mitigation interventions

**DOI:** 10.1093/tas/txac041

**Published:** 2022-04-08

**Authors:** Julia Q Fouts, Mallory C Honan, Breanna M Roque, Juan M Tricarico, Ermias Kebreab

**Affiliations:** 1 Department of Animal Science, University of California, Davis, Davis, CA 95616, USA; 2 FutureFeed Pty Ltd Townsville, QLD 4810, Australia; 3 Innovation Center for US Dairy, Rosemont, IL 60018, USA

**Keywords:** enteric methane, mitigation, ruminants

## Abstract

Mitigation of enteric methane (CH_4_) presents a feasible approach to curbing agriculture’s contribution to climate change. One intervention for reduction is dietary reformulation, which manipulates the composition of feedstuffs in ruminant diets to redirect fermentation processes toward low CH_4_ emissions. Examples include reducing the relative proportion of forages to concentrates, determining the rate of digestibility and passage rate from the rumen, and dietary lipid inclusion. Feed additives present another intervention for CH_4_ abatement and are classified based on their mode of action. Through inhibition of key enzymes, 3-nitrooxypropanol (3-NOP) and halogenated compounds directly target the methanogenesis pathway. Rumen environment modifiers, including nitrates, essential oils, and tannins, act on the conditions that affect methanogens and remove the accessibility of fermentation products needed for CH_4_ formation. Low CH_4_-emitting animals can also be directly or indirectly selected through breeding interventions, and genome-wide association studies are expected to provide efficient selection decisions. Overall, dietary reformulation and feed additive inclusion provide immediate and reversible effects, while selective breeding produces lasting, cumulative CH_4_ emission reductions.

## INTRODUCTION

Crucial goals for the 21st century include the mitigation of climate change and the provision of food to a growing population. Livestock agriculture contributes to both goals through continuous improvements in reproductive and nutritional efficiencies (Capper et al., 2009; Capper, 2011). However, more drastic efforts are needed to decrease greenhouse gas (GHG) emissions within a reasonable timeline (Beauchemin et al., 2020). Public concern for the environmental impact of animal agriculture is also increasing, applying additional pressure to reduce emissions. Globally, the livestock sector contributes 9–25% of anthropogenic GHG emissions, with the range in values attributed to different models and emission sources (Gerber et al., 2013; Rivera-Ferre et al., 2016). Ripple et al. (2014) reported that ruminants contribute 11.6% of global anthropogenic emissions when considering GHGs on a carbon dioxide (CO_2_) equivalence scale. Greenhouse gas emissions from livestock production include nitrous oxide (N_2_O) from manure application and nitrogenous fertilizers; CO_2_ from fossil fuels and land-use changes; and methane (CH_4_) from enteric fermentation and manure decomposition (USEPA, 2021). In November 2021, the Global Methane Pledge was launched, with 105 countries committed to reducing CH_4_ emissions by 30% over the next 10 years from 2020 levels (European Commission, 2021). Of total CH_4_ emissions in the United States, 27% are attributed to enteric fermentation (USEPA, 2021). Thus, practical approaches to reducing enteric CH_4_ position livestock agriculture as a key player in climate change mitigation.

### Review Objective

The objective of this review is to present an update on enteric CH_4_ mitigation interventions, including dietary reformulation, feed additive inclusion, and selective breeding. Enteric CH_4_ emissions are reported as production (g CH_4_/d), yield (g CH_4_/kg dry matter intake [DMI]), and intensity (g CH_4_/kg animal product) and are determined via live animal measurements or estimated through modeling. In addition to CH_4_ reductions from each intervention, impacts on rumen fermentation patterns, animal health and productivity, and net GHG emissions require consideration. When available in the literature and applicable to the intervention, a description is provided on the following: mode of action, efficacy (based on in vivo meta-analyses), inclusion rates, moderating variables, current or potential use in the field, and upstream and downstream GHG emissions. Because several interventions targeting enteric CH_4_ emissions are designed for intensive production systems, extra attention is placed on the potential for adoption in extensive systems.

### Upstream and Downstream GHG Emissions

**Figure 1.**
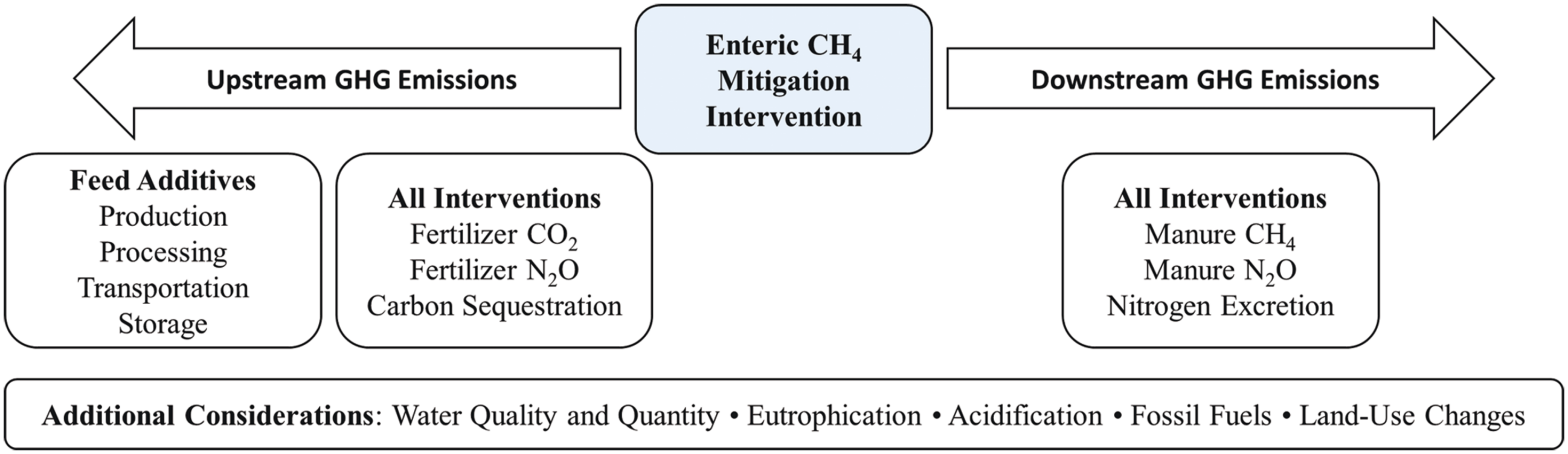
Considerations for determining the impact of enteric methane (CH_4_) mitigation interventions on environmental impact categories, including greenhouse gas (GHG) emissions (carbon dioxide (CO_2_) and nitrous oxide (N_2_O)). Adapted from FAO (2020) .

Before widespread adoption of enteric CH_4_ mitigation interventions, holistic and systematic approaches are needed to quantify the impact on net GHG emissions. Enteric CH_4_ emission reduction does not directly translate to a positive climate impact; upstream and downstream consequences on N_2_O, CO_2_, and CH_4_, as well as other environmental considerations, influence the full impact of an intervention (Fig. 1). Life cycle assessments (LCAs) quantify environmental impact categories such as GHG emissions, water quality and quantity, eutrophication, acidification and fossil fuel use of a given practice or product (Fig. 1) (FAO, 2020). The overall environmental impact of an intervention will depend on the production system with which it is integrated. Extensive systems provide the advantage of greater potential for CO_2_ sequestration, whereas intensive systems usually report lower enteric CH_4_ intensity. In general, feed additive LCAs should quantify GHG emissions associated with the production (cultivation and harvest or chemical synthesis), processing, transportation, and storage (FAO, 2020), as well as impacts on nitrogen excretion and manure CH_4_ and N_2_O (Fig. 1). The current review attempts to indicate, where appropriate, unique benefits, detriments, and opportunities for downstream and upstream GHG emissions (expressed as CO_2_ equivalents) for the interventions to highlight potential impacts beyond enteric CH_4_.

### Enteric Methane Production

Microbial action in the reticulorumen allows for cattle to utilize crops, crop residues, and by-products unfit for human consumption by converting structural carbohydrates and nonprotein nitrogen into nutrient-dense foods (Oltjen and Beckett, 1996; Newbold and Morales, 2020). However, rumen fermentation also produces enteric CH_4_, resulting in climatic implications and a loss of energy from the animal that could have been partitioned toward growth or production (Johnson and Johnson, 1995). Understanding the purpose and mechanism of CH_4_ production is crucial for the development of mitigation interventions.

Methane is produced in the reticulorumen by methanogenic archaea and released via eructation to maintain the negative redox potential favorable for the growth of strict anaerobic ruminal microorganisms (Van Soest, 1994; Morgavi et al., 2010). Methanogenesis is the main mechanism for removing hydrogen (H_2_), which impedes carbohydrate fermentation and fiber degradation through the accumulation of NADH (Morgavi et al., 2010). Methanogenic archaea are characterized based on their preference for substrate utilization: hydrogenotrophs produce CH_4_ with CO_2_ as the carbon source and H_2_ or formate as the electron donor; methylotrophs utilize methyl groups; and acetoclastic methanogens obtain carbon from acetate (Morgavi et al., 2010; Mizrahi et al., 2021). Current literature indicates that *Methanobrevibacter* spp. are the predominant rumen methanogens and responsible for the majority of CH_4_ production (Janssen and Kirs, 2008; Pitta et al., 2018). Although an evolving area of research, the hydrogenotrophic pathway (CO_2_ + 4H_2_ → CH_4_ + 2H_2_O) is considered the predominant mechanism for rumen CH_4_ production (Morgavi et al., 2010). Formed as byproducts during the production of volatile fatty acids (VFAs), H_2_ and CO_2_ are used by methanogenic archaea for growth and methanogenesis (Van Soest, 1994; Ungerfeld and Kohn, 2006; Morgavi et al., 2010). Although methanogens are responsible for CH_4_ production, interactions with other microbial populations impact their function (Martínez-Álvaro et al., 2020). For example, under a symbiotic relationship, protozoa provide excess H_2_ to methanogenic archaea via interspecies transfer (Morgavi et al., 2010).

## DIETARY REFORMULATION

Dry matter intake is the predominant predictor of enteric CH_4_ emissions due to the direct relationship between feedstuffs and microbial capacity for methanogenesis (Niu et al., 2018). However, feed composition and quality influence the microbial population, impacting the fate of H_2_ and overall fermentation patterns in the rumen.

### Forage-to-Concentrate Ratio

A decreased forage-to-concentrate ratio reduces CH_4_ emissions by shifting rumen fermentation patterns and proportions of VFAs. Forages are composed of structural carbohydrates, including cellulose and hemicellulose, which favor the production of acetate and butyrate, resulting in more H_2_ available for methanogenesis (Ungerfeld, 2020). Conversely, concentrates consist of nonstructural carbohydrates, including starch, and increase propionate concentration (Agle et al., 2010). Propionate production requires reducing equivalents, making it an alternative H_2_ sink to methanogenesis (McAllister and Newbold, 2008). As a precursor for glucose and lactose, propionate uptake of H_2_ in ruminants also increases the utilization of metabolic energy compared to CH_4_ eructation (Johnson and Johnson, 1995; Newbold et al., 2005). Additionally, starch decreases rumen pH (unfavorable for methanogens), fiber digestibility, and H_2_ available for methanogenesis (Van Kessel and Russell, 1996).

Quantifying the optimal forage-to-concentrate ratio is difficult, as it varies depending on diet composition, animal type, and physiological state of the animal. Van Gastelen et al. (2019) investigated the effects of decreasing the forage-to-concentrate ratio using 24 studies. With an average increase of 386 g/kg DM in concentrates, CH_4_ yield decreased 26% in beef cattle, 14% in dairy cattle, and 6% in sheep. Methane intensity also decreased in each animal type: 31% in beef cattle, 27% in dairy cattle, and 10% in sheep. Additionally, increasing inclusions of dry-rolled corn from 225 to 838 g/kg DM in beef steers resulted in a quadratic decrease in CH_4_ and increase in efficiency of conversion from digestible to metabolizable energy (Fuller et al., 2020). Also, four in vivo studies reported an increase in milk yield from increasing concentrate inclusion (Agle et al., 2010; Aguerre et al., 2011; Olijhoek et al., 2018).

Although decreasing the forage-to-concentrate ratio is a well-known strategy for reducing enteric CH_4_, the adoption potential is limited. Grains are widely used in intensive systems, and excessive dietary starch results in laminitis, milk fat depression, and subacute ruminal acidosis through the accumulation of lactic acid. Propionate harnesses more energy from glucose than other VFAs, but excessive propionate can lead to reduced intake, as described by the hepatic oxidation theory (Allen et al., 2009), and milk fat depression (Agle et al., 2010; Olijhoek et al., 2018; Ungerfeld, 2020).

Compared to intensive systems, grazing ruminant diets have a greater capacity for increased concentrates, with the effect on enteric CH_4_ emissions largely depending on the baseline intake of quality herbage (Lovett et al., 2005; Jiao et al., 2014; Muñoz et al., 2015; Van Wyngaard et al., 2018a, 2018b). For example, in low to medium quality pasture for dairy cattle, increasing concentrate inclusions (0 g/kg DM, 281 g/kg DM, 461 g/kg DM) linearly increased CH_4_ production and decreased CH_4_ yield and intensity (Van Wyngaard et al., 2018b). However, on highly digestible pasture, an average of approximately 50 g/kg DMI and 230 g/kg DMI of concentrates increased CH_4_ production with no effect on CH_4_ yield or intensity from grazing dairy cattle (Muñoz et al., 2015). Generally, dietary concentrate inclusion will have a greater chance of reducing enteric CH_4_ when the base diet is composed of low-quality herbage (Zubieta et al., 2021).

Enteric CH_4_ reductions from concentrate inclusion should be compared to the GHG emissions associated with increased fertilizer use and decreased soil carbon sequestration from the conversion of pastureland to cropland (Petersen et al., 2013; Gutiérrez-Peña et al., 2019). Elevated dietary concentrates can also increase nitrogen losses (Condren et al., 2019; Molossi et al., 2020) and water usage (Pereira et al., 2018; Molossi et al., 2020), potentially straining future water sources. Published LCAs report contrasting impacts of concentrate inclusion in grazing ruminant diets on net GHG emissions (Gutiérrez-Peña et al., 2019; Molossi et al., 2020; McGee et al., 2022); LCA results should be cautiously interpreted, as they are dependent on the region, system boundaries, modeling approaches, and pasture quality.

An additional concern of concentrate inclusion is the sourcing of crops away from human consumption (Molossi et al., 2020; McGee et al., 2022) while an advantage of forage-fed ruminants is the utilization of structural carbohydrates indigestible by humans. Furthermore, the practicality of increasing dietary concentrates depends on the regional price and quality of available grains and forages.

### Forage Quality

Forages are a major feed source for ruminants, and improving forage quality and digestibility is a feasible intervention for CH_4_ reduction (Hristov et al., 2013). Increased forage digestibility decreases CH_4_ intensity by enhancing the digestible energy available to the animal (Jung and Allen, 1995). As forages increase in maturity, more lignin forms on the cell wall, increasing cross-linkages and decreasing cell wall degradability and polysaccharide hydrolysis (Jung and Allen, 1995). Increasing maturity also results in fiber accumulation accompanied by a decline in soluble carbohydrates, protein, and fat. Although increased DMI is theoretically necessary to acquire nutrients from relatively lower digestible forages, physical fill limits intake and poor forage quality decreases the passage rate from the rumen (Allen and Mertens, 1988). Therefore, lower quality forages can increase CH_4_ yield and intensity due to decreased animal performance.

In dairy cattle, a 25% increase in grass silage or herbage digestibility resulted in decreased CH_4_ yield and intensity by 10% and 19%, respectively, due to increased passage rate from the rumen and animal productivity (Van Gastelen et al., 2019). However, the authors also report an 8% increase in CH_4_ production, which is most likely attributed to more substrates available for rumen microbes from increased DMI (Van Gastelen et al., 2019; Beauchemin et al., 2020). In all-forage beef cattle diets, a 33% increase in forage digestibility resulted in a 7% increase in CH_4_ production and no effect on CH_4_ yield (Van Gastelen et al., 2019). The discrepancy in CH_4_ emission reductions between animal types was due to the presence of concentrates in the dairy diets as opposed to beef and sheep diets (Van Gastelen et al., 2019).

In addition, impacts of forage digestibility in different geographical areas determine regionally specific interventions. For example, temperate regions primarily utilize C3 grasses and cold climate legumes, while tropical areas use C4 plants and warm climate legumes in ruminant diets. Archimède et al. (2011) conducted a meta-analysis on 22 studies with goats, sheep, and cattle with temperate and tropical forages. Production of CH_4_ was 10–17% lower for C3 grasses compared with C4 grasses, attributed to the higher lignin and neutral-detergent fiber (NDF) content and lower passage rate from the rumen of C4 grasses. Despite the apparent differences between C3 and C4 grasses, recent literature postulates similar enteric CH_4_ emissions given optimal management conditions (Ku-Vera et al., 2020a; Zubieta et al., 2021). For example, Archimède et al. (2018) reported similar CH_4_ emissions between sheep breeds that are suited to tropic environments fed C4 forages and those native to temperate climates fed C3 forages. Also, warm season legumes decrease CH_4_ by 20% compared to C4 grasses (Archimède et al., 2011), showing the possibility of replacing C4 grasses with legumes in warm climates (Hristov et al., 2013). One reason for reduced CH_4_ from legumes is the presence of secondary metabolites (Eugène et al., 2021).

Forage digestibility is also considered to be increased by the replacement of grass and legume silages with high quality corn silage containing a large proportion of starch (Hristov et al., 2013). For example, Hassanat et al. (2013) replaced alfalfa with corn silage at three levels of inclusion (0, 282, and 560 g/kg DM), finding that starch and apparent total-tract digestibility of DM, crude protein, and NDF increased with larger inclusion rates. As corn silage increased from 0 to 560 g/kg DM, CH_4_ yield decreased linearly with quadratic reductions in production and intensity (Hassanat et al., 2013). Corn silage also increased milk yield and protein, while decreasing milk fat. Interestingly, when corn silage increased to 282 g/kg DM, CH_4_ production, yield, and intensity either increased or remained constant, showing the need for high amounts of corn silage for an effective reduction. Uddin et al. (2020) also compared alfalfa and corn silage, each at two levels of NDF. Diets higher in corn silage decreased CH_4_ yield 8%, while production and intensity were more influenced by NDF than forage type. The relative NDF and digestibility of corn silage compared to grass and legume silages differs across regions and management practices, thus influencing the effect of a forage change on CH_4_ emissions.

In addition to considering respective digestibility values before replacing grass or legumes with corn silage, it is crucial to consider upstream and downstream GHG emissions prior to adoption. For example, manure CH_4_ is generally increased from the inclusion of corn silage (Hellwing et al., 2014; Eugène et al., 2021), which could overshadow the reductions in enteric CH_4_. Compared to corn production, legumes can decrease the input of nitrogenous fertilizers, allowing for the reduction of N_2_O emissions. Additionally, converting grassland for corn silage production can incur soil nitrogen and carbon losses (Vellinga et al., 2011; Eugène et al., 2021), and compensating for the GHG emissions from this land-use change with enteric CH_4_ reductions requires regional and temporal quantification (Vellinga et al., 2011). Corn silage production has also been shown to increase soil carbon loss compared to other crops (Poyda et al., 2019; Gamble et al., 2021). Therefore, quantifying the net GHG emission impact from a change in forage species is crucial prior to adoption.

Forage and pasture management also play a key role in reducing enteric CH_4_ emissions. In confinement-based systems, forage quality is improved through optimal harvest timing, silage preservation, and storage conditions (Hristov et al., 2013; Beauchemin et al., 2020). Pasture management in both temperate and tropical regions can decrease enteric CH_4_ by allowing for individual intake of high-quality forage through short sward height and a balance between herbage growth rate, ruminant stocking rate, and stocking density (Zubieta et al., 2021). Rotatinuous grazing is a management practice that considers sward structure along with animal consumption behavior and is shown to reduce CH_4_ intensity when considered per unit of land and animal product (Savian et al., 2018, 2021). Currently, a great amount of opportunity remains for more research to elucidate enteric CH_4_ mitigation and carbon cycles in pasture operations under various management conditions.

### Lipids

Lipids are hydrolyzed into fatty acids (Tamminga and Doreau, 1991) and are currently supplemented in diets to enhance energy density and utilization, increase milk yield, and manipulate milk’s fatty acid profile (Boadi et al., 2004). Lipids reduce enteric CH_4_ through several proposed mechanisms, such as providing an alternative H_2_ sink through bio-hydrogenation of unsaturated fatty acids, creating a shift to propionic production in the rumen, and directly inhibiting protozoa and methanogens (Yanza et al., 2020; Honan et al., 2021). Additionally, organic matter fermentation and fiber digestibility decrease when lipids replace carbohydrates, resulting in a reduction of both VFA production and methanogenesis (Johnson and Johnson, 1995; Haque, 2018).

A meta-analysis of seven studies showed 8–9% reduction in CH_4_ production and intensity when fat content increased, on average, from 25 to 64 g/kg DM in dairy cattle diets (Eugène et al., 2008). The authors also reported a 6.5% decrease in DMI without impacting milk production. Patra (2013) analyzed 29 studies from dairy and beef cattle, reporting a 15.1% decrease in CH_4_ emissions when lipid concentration increased from 20 to 60 g/kg DM. Lipid inclusion also increased propionate production, indicating a higher utilization of metabolic energy in the rumen (Patra, 2013).

Lipids have been found to decrease CH_4_ emissions in a dose–response manner; for every 1% increase in dietary fat content, CH_4_ yield decreased by 0.66–1.00 g/kg DM (Patra, 2013). However, excessive lipid supplementation poses challenges to cattle health and lactation performance (Hristov et al., 2013; Knapp et al., 2014), and milk production declines if fed over 60 g/kg DM of lipids (Patra, 2013). According to the NRC (2001), lipid content should not exceed 70 g/kg DM. When fed below the maximum threshold, Eugène et al. (2008) reported a 7% increase in feed efficiency.

Two types of lipids are considered the most effective in CH_4_ reduction: medium-chained fatty acids (MCFA) and polyunsaturated fatty acids (PUFA) (Rasmussen and Harrison, 2011; Patra, 2013). In a meta-analysis of 21 in vivo studies, Yanza et al. (2020) reported coconut oil to be the most consistent source of MCFA in CH_4_ reduction, resulting in 21% reduction in CH_4_ yield and a 28% decrease in CH_4_ production. The authors also determined 40 g/kg DM as the effective MCFA inclusion for CH_4_ reduction (Yanza et al., 2020). Wang et al. (2017) found two PUFA sources, safflower seeds and hemp, to be effective in CH_4_ abatement under in vitro conditions when supplied at 70 g/kg DM. A limited number of in vivo studies show the potential for PUFA in reducing CH_4_ emissions (Li et al., 2011; Bayat et al., 2017), and the risk of milk fat depression from feeding PUFA to dairy cattle must be considered (Rico and Harvatine, 2013).

The efficacy of lipids in CH_4_ reduction is dependent on the form, inclusion rate in the diet, fatty acid profile, and the basal diet (Eugène et al., 2008; Patra, 2013). In grazing pastures, canola oil spray increased dietary lipid inclusion by approximately 50 g/kg DM (compared to the control diet), decreasing CH_4_ production (11%) and yield (18%) from steers (Pinares-Patiño et al., 2016). Also, a 22 g/kg DM increase in dietary ether extract from whole cotton seeds (126 g/kg DM) for grazing dairy cattle initially decreased CH_4_ yield 14% but with a lack of persistency throughout seasons, indicating potential ruminal adaptation (Muñoz et al., 2021). Lipids can also reduce fiber digestibility, and more research is needed on delivery in pastures (Almeida et al., 2021). Because the predominant moderating variable for lipid efficacy is fiber concentration, lipids may be more practical for adoption by feedlots using low-fiber diets (Patra, 2013).

The negative impact of lipids on fiber digestibility could increase CH_4_ emissions from manure. Also, an LCA presented by Herd et al. (2014) demonstrated that lipid supplementation during the winter months in Australia would increase net GHG emissions, with manufacturing and transportation outweighing the 14% and 10.5% reduction in enteric CH_4_ yield for youngstock and adult beef cattle, respectively (Herd et al., 2014). However, ruminant consumption of lipids sourced from byproducts (Paula et al., 2019) has the added benefit of converting waste into animal protein (Salami et al., 2019).

## FEED ADDITIVES

Feed additive use is another area of active research for potential enteric CH_4_ reductions (Hristov et al., 2013; Honan et al., 2021). The present review categorizes feed additives as methanogenesis inhibitors or compounds that influence ruminal fermentation metabolic pathways.

### Methanogenesis Inhibitors

One of the most effective interventions for enteric CH_4_ reduction is direct inhibition of methanogenesis (Almeida et al., 2021). Depending on the magnitude of CH_4_ suppression (Ungerfeld et al., 2022) and the availability of alternative H_2_ metabolic pathways (Zhang et al., 2021), H_2_ emissions have been shown to increase from the direct prevention of the CH_4_ formation (Kinley et al., 2020; Roque et al., 2021). A meta-regression from Ungerfeld et al. (2022) found that the energy lost by H_2_ as a function of energy saved in CH_4_ reduction increased with the level of methanogenesis inhibition. Although Ungerfeld et al. (2022) reported no perceived effects from elevated H_2_ on rumen fermentation, the potential for interfering with digestion through inhibition of NADH oxidation should be further investigated. Two feed additives receiving attention for their effect on methanogenesis are 3-nitrooxypropanol (3-NOP) and macroalgae.

#### 3-Nitrooxypropanol.

The organic molecular compound 3-NOP reduces CH_4_ emissions with minimal impact on animal production parameters (Jayanegara et al., 2018; Kim et al., 2020). The mode of action of 3-NOP involves methyl-coenzyme M reductase (MCR), which is a Ni enzyme responsible for catalyzing the final step of CH_4_ formation from methyl-coenzyme M (Duin et al., 2016). The Ni in the MCR is bound in a tetrapyrrole derivative (F_430_), a co-factor that needs to be in the Ni(I) oxidation state for MCR to be active. 3-Nitrooxypropanol has a similar molecular shape to that of methyl-coenzyme M and inactivates MCR by oxidizing its active site Ni(I) (Duin et al., 2016). Once inhibited, MCR will not catalyze the final step in CH_4_ production.

Dijkstra et al. (2018) conducted a meta-analysis on 11 experiments with beef and dairy cattle supplemented with 3-NOP. A mean inclusion rate of 0.123 g/kg DMI reduced CH_4_ production by 32.5% and yield by 29.3% (Dijkstra et al., 2018). Methane production decreased by 1.0% to 3.0% for every increase in inclusion from the mean by 0.01 g/kg DMI. However, higher inclusions of 3-NOP are necessary for beef than dairy cattle (Dijkstra et al., 2018; Kim et al., 2020). The discrepancy between animal types is hypothesized to be from higher DMI in dairy cattle, resulting in more alternative H_2_ sinks and thus a lower concentration of methyl-coenzyme M and potential for MCR inhibition (Dijkstra et al., 2018). The effect of 3-NOP is also hindered by increased fiber content; for every 0.01 g/kg DM increase in NDF from its mean, 3-NOPs inhibitory effect on CH_4_ production decreased by 1.64% (Dijkstra et al., 2018). Jayanegara et al. (2018) evaluated 12 3-NOP experiments with dairy cattle, beef cattle, and sheep, finding that an inclusion rate of 0.100 g/kg DM decreased CH_4_ yield and intensity by 19.2% and 21.1%, respectively. In their meta-analysis, 3-NOP did not affect DMI or milk production, decreased VFAs, and increased milk fat concentration for dairy and feed conversion ratio in beef cattle (Jayanegara et al., 2018). Although several studies agree with no impact on milk production and increased milk fat concentration (e.g., Van Gastelen et al., 2020; Melgar et al., 2021), a recent meta-analysis with 14 in vivo studies reported a tendency for decreased milk yield with increasing levels of 3-NOP supplementation (Kim et al., 2020), highlighting the importance of the minimum effective inclusion and following the manufacturer’s recommendations.

As a water-soluble molecule that does not require active transport, 3-NOP appears to be metabolized quickly in the rumen (Duin et al., 2016). Therefore, 3-NOP should be continuously available in the feed (Reynolds et al., 2014; Hristov et al., 2015), whether mixed with the total mixed ration (TMR) or as part of a concentrate pellet (Van Wesemael et al., 2019). Because the continuous supply of 3-NOP to grazing ruminants is challenging, adoption of 3-NOP can occur more rapidly in confinement, intensive-based systems. Given the consistent and safe CH_4_ reductions from 3-NOP, research on the delivery method and efficacy of 3-NOP to grazing ruminants could add invaluable reduction potential. Additionally, 3-NOP cannot be implemented in organic production systems as it is a synthetic product.

In addition to consistent reductions in CH_4_, 3-NOP is predicted to reduce net GHG emissions in multiple regions and production systems. When CO_2_, N_2_O, and CH_4_ were considered in a cradle-to-farm gate LCA for the California dairy industry, the supply of 3-NOP (0.127 g/kg DM) resulted in an average reduction of 11.7% in net GHG emission intensity (Feng and Kebreab, 2020). Based on a partial LCA, the consumption of 3-NOP (0.086 g/kg DM) by lactating dairy cattle on two Australian dairy farms led to a 14% decrease in whole-farm GHG emission intensity (Alvarez-Hess et al., 2019). The greater GHG reduction in Australia compared to California is attributed to differences in GHG inventories for each region as well as the greater manure CH_4_ emissions in California.

In September 2021, the Chilean and Brazilian governments granted regulatory approval of Bovaer (Royal DSM, NL), the commercialized form of 3-NOP (DSM, 2021). Bovaer also received marketing approval for dairy cattle from the European Union in February 2022 (DSM, 2022). Approval of Bovaer from the Food and Drug Administration in the United States is still under consideration.

#### Macroalgae.

Macroalgae grow in either salt or fresh water and can contain sulfur-based amino acids, minerals, and carbohydrates (Makkar et al., 2016). Some macroalgae contain elevated levels of tannins or lipids (PUFA) which result in moderate CH_4_ reduction (Abbott et al., 2020); the most effective species are those containing halogenated CH_4_ analogs, such as bromoform (Machado et al., 2016; Abbott et al., 2020). Bromoform reduces CH_4_ by inhibiting the cobamide-dependent methyltransferase needed for methanogenesis (Wood et al., 1968).

A meta-analysis reviewing anti-methanogenic macroalgae with both modes of action concluded 36% CH_4_ yield reductions with no effects on DMI, average daily gain (ADG), milk yield, or milk components (Lean et al., 2021). *Asparagopsis* species (*A. taxiformis* and *A. armata*) are considered the most effective macroalgae due to high concentrations of bromoform (Machado et al., 2014; Kinley et al., 2016). Five in vivo studies are published using *Asparagopsis* in sheep (Li et al., 2016), dairy cattle (Roque et al., 2019a; Stefenoni et al., 2021), and beef steers (Kinley et al., 2020; Roque et al., 2021). Sheep fed *A. taxiformis* at 78.4 g/kg DM reduced CH_4_ yield by 80% with no effects on ADG or DMI (Li et al., 2016). Kinley et al. (2020) reported 98% less CH_4_ yield and a 22% increase in ADG in beef steers fed 3.7 g/kg DM *A. taxiformis.* Beef cattle fed 4.9–9.8 g/kg DM *A. taxiformis* over low, mid, and high forage diets showed reduced CH_4_ yields (67–83%) with decreasing forage levels and 7–14% increases in feed conversion efficiency (Roque et al., 2021). Methane yield reductions of 80% were reported in lactating dairy cows fed 5.0 g/kg DM, but the persistence of this reduction dropped in concert with declining bromoform in the *A. taxiformis* (Stefenoni et al., 2021). Furthermore, milk yield and energy corrected milk decreased, attributed to reduced DMI (Stefenoni et al., 2021). Roque et al. (2019a) showed similar results in lactating dairy cows fed 18.4 g/kg DM *A. armata*, with a 67% decrease in CH_4_ intensity along with reductions of 38% DMI and 12% milk production (Roque et al., 2019a). Rumen fermentation effects, such as total VFA production, are inconsistent. However, the reduction of acetate-to-propionate is consistently reported in vivo (Li et al., 2016; Kinley et al., 2020; Stefenoni et al., 2021). Additionally, only one study has showed long-term efficacy (21 weeks) (Roque et al., 2021), thus more long-term studies with greater animal numbers are needed. Furthermore, macroalgae studies should be conducted in pasture-based systems to assess impacts on health, production, and CH_4_ emissions.

Because anti-methanogenic effects are dependent on bromoform levels, feeding recommendations should be based on bromoform concentrations. Time of harvest, species, water conditions, processing, and storage influence bromoform concentrations (Makkar et al., 2016; Abbott et al., 2020; Stefenoni et al., 2021). However, bromoform is ozone-depleting and with poor handling can be released into the atmosphere. Muizelaar et al. (2021) attempts to determine the rate of bromoform transfer to milk from cows consuming *Asparagopsis*. While the results from this study are variable, bromoform was detected in one cow consuming *Asparagopsis* above 20 g/kg DM and was heavily feed restricted. Furthermore, Muizelaar et al. (2021) had no control group whereas Roque et al. (2019a) and Stefenoni et al. (2021) did include control groups and found trace amounts of bromoform in all milk samples tested. Both studies reported no significant differences in milk bromoform between cattle consuming *Asparagopsis* and the control diet. Muscle, fat, and organs from *Asparogopsis* fed animals have been tested for bromoform residues to which none has been found (Li et al., 2016; Kinley et al., 2020; Roque et al., 2021). Macroalgae studies have reported elevated milk iodine concentrations (Antaya et al., 2015; Stefenoni et al., 2021), which may pose public health risks (Makkar et al., 2016; Zimmermann et al., 2005). Overall, the greatest barriers to macroalgal commercialization include large-scale production (Makkar et al., 2016) and regulatory approval (Honan et al., 2021).

Ocean-based macroalgae production provides the added environmental benefits of CO_2_ sequestration, reduction of ocean acidification, and water quality improvement in areas facing eutrophication (Krause-Jensen and Duarte, 2016; Duarte et al., 2017; Jagtap and Meena, 2022). When considering offshore production, the supplementation of *A. taxiformis* (0.0715 g/kg DM) in Australian feedlots was projected to reduce net GHG emissions by 1–4% for the country’s beef industry by 2030 compared to 2018 (Ridoutt et al., 2022). However, without the combination of rapid adoption, high efficacy in enteric CH_4_ reduction, and increased ADG, industry emissions were predicted to increase (Ridoutt et al., 2022).

An alternative to harvesting macroalgae from the ocean is the implementation of land-based production. Nilsson and Martin (2022) performed an LCA on a future, land-based *A. taxiformis* production system located in Sweden. Although the system boundaries did not include the reduction of enteric CH_4_, salt input was the largest contribution to GHGs, with the source of salt and rate of water recycling as ideas to reduce this impact. If growing *A. taxiformis* in a land-based system in the northern part of the world, more energy may be needed to imitate the tropical environment required for growth (Nilsson and Martin, 2022). However, the more temperate *A. armata* has been found as far north as Ireland and may have greater opportunity to be grown in temperate environments.

### Rumen Environment Modifiers

Modification of the rumen environment to create unfavorable conditions for methanogens presents another intervention for CH_4_ mitigation. Such modifications include the provision of alternative H_2_ sinks or suppression of the activity of microbes involved in a symbiotic relationship with methanogens. Nitrate and secondary compounds, including essential oils (EO) and tannins, are described in the present review as rumen environment modifiers.

#### Nitrate.

Nitrate is a polyatomic inorganic ion that provides an alternative H_2_ sink in the rumen, leading to a decrease in CH_4_. Once in the rumen, nitrate reduces to nitrite (NO_3_^−^ + H_2_ → NO_2_^−^ + H_2_O), which reduces to ammonia (NO_2_^−^ + 3H_2_ + 2H^+^ → NH_4_^+^ + 2H_2_O). Compared to the main pathway of methanogenesis (CO_2_ + 4H_2_ → CH_4_ + 2H_2_O), the reduction of nitrate and nitrite has greater Gibbs free energy changes, making nitrate thermodynamically more favorable than methanogenesis for H_2_ (Ungerfeld and Kohn, 2006). As explained by Lee and Beauchemin (2014), one mole of nitrate (100 g) prevents the production of one mole of CH_4_ (26 g). Nitrate has also been shown to reduce populations of methanogens through slight nitrite toxicity (Zhou et al., 2012).

A meta-analysis by Lee and Beauchemin (2014) compiled eight in vivo nitrate studies in dairy cattle, beef cattle, sheep, and goats, reporting a linear reduction in CH_4_ yield and consistent efficacy from nitrate supplementation. Feng et al. (2020) conducted a meta-analysis with 24 in vivo experiments to uncover the source of variability in the effect of nitrate on CH_4_ production. The authors found that a mean nitrate inclusion rate of 16.7 g/kg DMI reduced CH_4_ production by 13.9% and CH_4_ yield by 11.4%. Nitrate supplementation also had no effect on milk yield, milk composition, DMI, or nutrient digestibility (Feng et al., 2020). Additionally, cattle type affected CH_4_ yield, with a 20.4% reduction for dairy and 10.1% for beef cattle, due to higher feed intake in dairy cattle and the greater use of slow-release nitrates in beef cattle (Feng et al., 2020).

Nitrate’s ability to reduce CH_4_ is affected by its inclusion rate (Lee and Beauchemin, 2014; Feng et al., 2020). Every increase of nitrate by 1 g/kg DM from 16.7 g/kg DM resulted in a 0.904% decrease in CH_4_ yield (Feng et al., 2020). Olijhoek et al. (2016) demonstrated nitrate’s dose-dependent behavior, finding that CH_4_ yield decreased by 6%, 13%, and 23% for low (5.3 g/kg DM), medium (13.6 g/kg DM), and high (21.1 g/kg DM) nitrate supplemented diets, respectively.

As a nonprotein source of nitrogen, nitrate is an option for supplementing low-protein diets and is a suggested feed additive to replace urea for CH_4_ reductions (Lee and Beauchemin, 2014). When replacing protein meals on California dairy farms, an LCA demonstrated that nitrate supplemented to the whole herd at 16.7 g/kg DM had a 4.96% reduction in net GHG emission intensity (Feng and Kebreab, 2020). However, the magnitude of reduction in GHG emission intensity associated with crop production for protein meals is surpassed by the emissions from nitrate production (Feng and Kebreab, 2020).

In grazing systems, nitrate is useful during seasons with naturally lower protein content (Callaghan et al., 2014). In beef steer diets, the supplementation of encapsulated nitrate for 13 months (66.6 g/kg DM during dry season, 93.3 g/kg DM during rainy season, 32.5 g/kg DM on finished diet) resulted in 18.5% decrease in CH_4_ yield (g CH_4_/kg forage DMI) compared to a urea supplemented diet (Granja-Salcedo et al., 2019). The authors also found no microbial adaptation and an increase in ADG. For dairy cattle, two studies reported no significant decreases in CH_4_ emissions from nitrate supplementation on pasture, predominantly due to elevated nitrate levels in the control diet (Van Wyngaard et al., 2018c, 2019). Overall, more research is needed on the safety and efficacy of CH_4_ reduction from nitrates in low-protein grazing diets.

Additionally, a major challenge with nitrates is the potential for nitrite toxicity through increased methaemoglobin, a type of hemoglobin incapable of releasing oxygen to tissues (Lee and Beauchemin, 2014). Options to manage nitrite toxicity are not widely available and are more difficult to address in extensive systems. Nitrate has also been shown to increase H_2_ emissions from the animal, representing another form of energy loss (Lee et al., 2017; Almeida et al., 2021). Gradual rumen acclimation and encapsulated nitrate can prevent nitrite toxicity and elevated H_2_ (Lee and Beauchemin, 2014; Almeida et al., 2021).

#### ***Essential oil*** s.

These contain volatile, lipophilic secondary metabolites (Benchaar and Greathead, 2011; Ugbogu et al., 2019), and different hypotheses have been put forward to explain the mode of action in CH_4_ mitigation. By accumulating in the lipid bilayer and cytoplasm, EO may disrupt microbial functioning (Benchaar and Greathead, 2011; Ugbogu et al., 2019). Essential oils are also thought to increase propionate concentrations, decreasing the availability of H_2_ for CH_4_ production (Ugbogu et al., 2019).

Khiaosa-ard and Zebeli (2013) conducted a meta-analysis with 28 studies on EO in beef cattle, dairy cattle, and sheep. In beef cattle, CH_4_ production decreased 12% by supplementing 0.25 g EO/kg DM to the diet with a more pronounced reduction compared to other ruminants due to a lower rumen pH from a high concentrate diet (Khiaosa-ard and Zebeli, 2013). The influence of pH on the efficacy of EO warrants the quantification of CH_4_ reductions at different ruminant life stages and diet compositions. A meta-analysis by Torres et al. (2021) found no effects on CH_4_ production or beef cattle performance when comparing EO and the monensin additive, making EO a possible alternative to ionophores. However, EO increased the risk of liver abscesses in beef cattle (Torres et al., 2021). Interpretation of meta-analyses combining studies on various EO must be met with caution, and more meta-analyses are needed on individual EO.

In addition to single EO added to the diet, EO can be provided to ruminants through commercial blends. Mootral (Mootral S.A., Rolle, Switzerland), a commercial blend containing garlic and bitter orange extract, decreased CH_4_ in vitro by altering the rumen archaeal community (Eger et al., 2018). When supplemented at 1.58 g/kg DM, Mootral decreased CH_4_ yield from beef cattle by 23.2%, but this effect was only observed in the final and 12th week of a trial with a small number of animals (Roque et al., 2019b). No negative effects were found on DMI, ADG, or feed conversion efficiency. An on-farm trial supplied Mootral at an inclusion of 1.2 g/kg DM for Jersey cattle and 0.64 g/kg DM for Holstein cattle, finding a 38.3% and 20.7% decrease in CH_4_ concentration (ppm), respectively (Vrancken et al., 2019). Future commercial Mootral trials should quantify CH_4_ emissions as production (g/d) for better comparison to other mitigation interventions. Also, due to the small number of animals in a limited number of published studies, more data is needed for a conclusive statement on the efficacy of Mootral in enteric CH_4_ mitigation.

Agolin Ruminant (Agolin S.A., Bière, Switzerland) is a commercial blend with coriander seed oil, eugenol, and geranyl acetate (Belanche et al., 2020). Belanche et al. (2020) conducted a meta-analysis on Agolin Ruminant with eight studies and reported an average decrease in CH_4_ production (8.8%), yield (12.9%), and intensity (9.9%) in trials longer than 4 weeks. A recent in vivo study on Agolin Ruminant reported a decrease in CH_4_ intensity with no impact on production parameters, CH_4_ production, or CH_4_ yield (Carrazco et al., 2020). The effect on CH_4_ intensity resulted from a numeric, nonsignificant decrease in CH_4_ production and increase in energy corrected milk yield (measured immediately following CH_4_ sampling). In contrast, energy corrected milk yield measured throughout the entire study numerically decreased from Agolin Ruminant, so the effects on CH_4_ intensity from Carrazco et al. (2020) could be due to chance. Also, due to the limited number of published studies, more research is needed to understand the dose–response and overall effects of Mootral and Agolin Ruminant. Although commercial products have the benefit of controlling and stabilizing the EO composition, the sourcing of EO, forming encapsulated pellets, powders, or liquids, and distributing the product incurs GHG emissions that need to be compared to enteric CH_4_ reductions.

The efficacy of EO depends on dietary inclusion rate and EO composition, which varies between and within plant species, different plant parts, and varying harvesting methods (Cosentino et al., 1999). Torres et al. (2021) reported inclusion rates ranging from 0.05 to 0.50 g/kg DM, and Khiaosa-ard and Zebeli (2013) used a mean of 0.10 g/kg DM in their analysis. Relatively low inclusion rates of EO are more consistent in CH_4_ reduction and less detrimental to cattle health and total VFA production (Patra and Yu, 2012; Khiaosa-ard and Zebeli, 2013).

Essential oils can be incorporated in confinement and pasture-based systems (Ku-Vera et al., 2020b). For research purposes, EO have been delivered in pastures in extracted forms (Flores et al., 2013; Beck et al., 2017; Teobaldo et al., 2020), but providing pre-processed supplements to grazing ruminants is challenging in practice. Although grazing systems can incorporate EO-containing plants for ruminant consumption and added biodiversity, regionally based research is needed on CH_4_ results from in vivo trials using plants containing EO across ruminant types and breeds. Also, the composition and variability of EO in plants should be tracked over time. Additional challenges include the broad antimicrobial activity of EO that could impact microbes beneficial to rumen health (Ku-Vera et al., 2020b) and the need for in vivo dose–response studies for proper implementation in the industry.

#### Tannins.

 These are water-soluble, polyphenolic plant secondary compounds that have been shown to reduce enteric CH_4_ (Jayanegara et al., 2012). Hydrolysable tannins directly inhibit methanogens but can be toxic to ruminants (Goel and Makkar, 2012), while condensed tannins are more heavily investigated for CH_4_ mitigation. Sources of condensed tannins recently studied include *Leucaena leucocephala* forage (Piñeiro-Vázquez et al., 2018; Montoya-Flores et al., 2020), *Acacia mearnsii* extract (Alves et al., 2017; Denninger et al., 2020), and grape marc (Moate et al., 2020). Hypothetical mechanisms of CH_4_ reduction from condensed tannins include: binding to proteins, carbohydrates, and microbial enzymes; providing an alternative H_2_ sink; and interrupting interspecies transfer of H_2_ (Tavendale et al., 2005; Naumann et al., 2017; Ku-Vera et al., 2020b).

A meta-analysis by Orzuna-Orzuna et al. (2021) compiled 32 studies to evaluate the effects of tannins (condensed, hydrolyzed, and mixed) on CH_4_ emissions in beef cattle. With an average inclusion rate of 14.6 g/kg DM, the production and yield of CH_4_ decreased by 10 and 5.9%, respectively (Orzuna-Orzuna et al., 2021). The authors found no effects on ADG, DMI, or feed efficiency. Using 84 studies, Yanza et al. (2021) conducted a meta-analysis on hydrolyzed, condensed, and mixed tannins supplied to cattle, sheep, and goats. Methane yield decreased linearly as tannin inclusion rate increased from 0 to 140 g/kg DM, along with a linear decrease in fat and protein corrected milk for dairy ruminants (Yanza et al., 2021). Results from the two meta-analyses elucidate the inconsistent effects on protein digestibility. Orzuna-Orzuna et al. (2021) report a shift from urinary nitrogen to fecal nitrogen, which could reduce available nitrogen for N_2_O emissions. However, Yanza et al. (2021) found no impact on urinary nitrogen and an increase in fecal nitrogen from decreased protein digestibility. Thus, more research is needed on the downstream effects of tannin supplementation on manure N_2_O emissions.

In parallel with other plant-sourced compounds, the effects of tannins on CH_4_ emissions depend on a combination of type, inclusion rate, and source (Jayanegara et al., 2012; Aboagye and Beauchemin, 2019). Relative thresholds of tannin inclusion have been approximated by Orzuna-Orzuna et al. (2021) who demonstrated that exceeding 12 g/kg DM negatively impacts DM and NDF digestibility and exceeding 50 g/kg DM reduces DMI, compromising feed conversion efficiency. While remaining mindful of those higher inclusion rates, enteric CH_4_ emissions tend to be reduced more consistently as tannin concentrations increase; the minimum for detecting consistent reductions is 20 g/kg DM (Jayanegara et al., 2012). Thus, more research is needed on the appropriate inclusion rate of tannins that effectively mitigate emissions without impeding feed digestibility.

Two options for dietary tannin inclusion are available in both temperate and tropical regions: extracted tannin supplements and tanniferous forages (Aboagye and Beauchemin, 2019). Extracted tannin supplements can be added to the TMR in confined feeding production systems, while tanniferous forages can be implemented in grazing systems. Grape marc, a waste product associated with winemaking, has the potential to decrease GHG emissions when added to ruminant diets as a tannin source (Muhlack et al., 2018). Trees containing tannins can be grown with forages and grazing ruminants in a silvopasture production system. If well-managed, silvopasture systems could offset enteric CH_4_ through increased CO_2_ sequestration, leading to decreased net GHG emissions (Oliveira Resende et al., 2020). Although an in-depth analysis of silvopasture is beyond the scope of this review, future work should characterize its effects on GHG emissions. Also, feeding tannin-containing plants must consider the effects on CH_4_ from other potentially present secondary metabolites (Jayanegara et al., 2012). Additional benefits of feeding tannins are its antiparasitic properties (Naumann et al., 2017), decreased prevalence of bloating, and reduced nitrogen excretion when dietary protein is excessive (Hristov et al., 2013). However, tannins can decrease fiber digestibility, palatability, DMI, and protein digestibility in crude protein-limited diets (Naumann et al., 2017).

## SELECTIVE BREEDING

Selective breeding provides the potential for long-term CH_4_ emission reductions that are sustained and accumulated over generations (Wall et al., 2010). Breeding interventions can be implemented in both intensive and extensive production systems (Beauchemin et al., 2020). Also, assisted reproductive technologies increase the rate of genetic change (Moore and Hasler, 2017). Due to the long-term nature of selective breeding, data from genetic studies relating to enteric CH_4_ emissions over the last decade are beginning to be understood and represented in the literature (Dillon et al., 2021; Manzanilla-Pech et al., 2022). Direct selection involves breeding decisions based on CH_4_ traits, while indirect selection breeds for animals with traits assumed to be correlated with CH_4_ emissions. Genomic selection is a newer approach to direct and indirect selective breeding and has the potential for future CH_4_ mitigation.

### Direct Selection

Direct selection against CH_4_ involves selecting low-emitting animals for breeding based on phenotype using emission measurements. Methane emissions vary between breeds, individual animals, and throughout the animal’s lifetime (De Haas et al., 2011). Heritability estimates are used to quantify the magnitude by which CH_4_ emissions are influenced by the genome, ranging from 0.12 to 0.45 (Dillon et al., 2021), and are proposed for sheep (Pinares-Patiño et al., 2013), dairy cattle (Lassen and Difford, 2020), and beef cattle (Manzanilla-Pech et al., 2020). Low to moderate heritability estimates suggest that CH_4_ emissions are partially controlled by the genotype, allowing for some degree of reduction through direct selection (Hayes et al., 2016).

Although limited studies are available for breeding against CH_4_ emissions, Pickering et al. (2015) predicted up to 25% reduction in CH_4_ yield from the selection of low emitters. The timeframe of the reduction is unclear and depends on the genetic mechanisms influencing CH_4_, as well as the ruminant type. If implemented in Dutch dairy cattle, De Haas et al. (2021) predicted that the selection of low emitters will reduce CH_4_ intensity by 24% over the next 30 years. Direct selection of sheep with low and high CH_4_ yield in New Zealand created two divergent progeny lines, resulting in an average difference of 10–12% in CH_4_ yield over 10 years (Rowe et al., 2019). Significant differences in CH_4_ yield between the divergent lines have been reported from controlled environments (Pinares-Patiño et al., 2013) and under grazing conditions (Jonker et al., 2017). When manure CH_4_ and N_2_O and urine N_2_O were considered, sheep from the low-CH_4_ yield line emitted 8% less GHG emissions compared to the high-CH_4_ yield line sheep across autumn and winter seasons (Jonker et al., 2019). However, research and development of direct selection is in the early stages, as well as the understanding of upstream and downstream consequences on net GHG emissions.

Despite the promising long-term reductions, accuracy and implementation of direct selection require CH_4_ emission data from thousands of animals, with the recommendation of 12,000 to 25,000 dairy cattle (De Haas et al., 2021). Furthermore, breeding decisions on farms require multiple, direct measurements of CH_4_ emissions from each animal (Lassen and Difford, 2020), which is impractical and expensive (Pickering et al., 2015). Additional challenges for successful direct selection are the determination of which CH_4_ trait to select against (production, yield, or intensity) and the relationship to other beneficial traits for appropriate inclusion into balanced selection indices (Lassen and Difford, 2020; De Haas et al., 2021).

### Indirect Selection

Indirect selection is based on reducing the impact of CH_4_ per unit of product coming from animal agriculture (CH_4_ intensity) through improved reproductive and nutrient-use efficiency (Pickering et al., 2015; Dillon et al., 2021). Knapp et al. (2014) determined that additive genetic selection for indirect traits, including milk yield, feed efficiency, heat-stress tolerance, and disease resistance, can reduce CH_4_ intensity by 9–19% at the animal and herd levels. Although potentially more practical for use on farms than direct selection, the correlation between CH_4_ emissions and selected traits warrants further research.

Feed efficiency is an indication of the ability to acquire nutrients from feed (Løvendahl et al., 2018) and presents a promising option for indirect selection of reduced CH_4_ (Pickering et al., 2015; Dillon et al., 2021). Residual feed intake (RFI) is the difference between actual and expected feed intake; a lower RFI indicates higher feed efficiency. Three theories for the reduction of CH_4_ from low RFI are presented by Basarab et al. (2013): lower feed intake for the desired level of milk or meat production, lower rumen retention time, and an increase in the acetate-to-propionate ratio. However, the relationship between RFI and CH_4_ is inconsistent. For example, De Haas et al. (2011) found ruminants with lower RFI had lower predicted CH_4_ production, while Flay et al. (2019) determined that low CH_4_ does not always indicate higher feed efficiency and could reflect a low ability to digest fiber. Flay et al. (2019) also reported improved digestion from greater feed efficiency, resulting in higher CH_4_ yield in heifers. Therefore, selecting for feed efficiency may not result in lower enteric CH_4_ emissions if CH_4_ yield increases and offsets the expected reduction from less DMI (Flay et al., 2019). Also, the determination of RFI requires DMI measurements, which is challenging in a commercial setting, especially in pasture-based systems (Beauchemin et al., 2020).

Relatively lower RFI ruminants are considered to produce the same amount of animal product with lower DMI (Herd et al., 2003; Potts et al., 2015), which can reduce the GHG emission burden of purchased feed compared to ruminants with higher RFI. Additionally, ruminants with low RFI may produce manure with less volatile solids and CH_4_ emissions during storage (Hansen et al., 2021), as well as less overall manure output (Connor et al., 2013).

### Genomic Selection

Genomic selection is an approach to either directly selecting for reduced CH_4_ traits or indirectly selecting for correlated traits. Before implementation, genomic selection requires CH_4_ emission measurements and genotypes from a large reference population to conduct genome-wide association studies (GWAS) (Hayes et al., 2016). Based on GWASs, the association between single-nucleotide polymorphisms (SNPs) and CH_4_ traits can be used in genomic estimated breeding values (GEBVs) to inform breeding decisions in the target population (Hayes et al., 2016).

Genome-wide association studies highlight the need to consider genetic correlations between CH_4_ traits and production characteristics. For example, GWASs from 1,020 Angus beef cattle showed significant associations for CH_4_ production on chromosomes 4, 14, and 20 (Manzanilla-Pech et al., 2016), which are also associated with ADG, carcass weight, and weight, respectively (Lindholm-Perry et al., 2012; Bolormaa et al., 2013). Additionally, Manzanilla-Pech et al. (2022) conducted GWASs on 1,962 Danish Holstein dairy cattle and 38,253 SNPs, finding strong associations for CH_4_ production on chromosome 13 and production, yield, and intensity on chromosome 26. Manzanilla-Pech et al. (2022) referenced a few other papers that utilized GWAS for SNP associations of CH_4_ emissions (Manzanilla-Pech et al., 2016; Pszczola et al., 2018; Calderón-Chagoya et al., 2019) to conclude a reliable association for CH_4_ production on chromosome 13. However, the authors emphasize the limitations in interpretation due to small sample sizes. Also, genetic correlations between CH_4_ traits are limited, so the trait definition (and how it is determined) will impact results.

Currently, GEBV accuracies are low due to limited data on CH_4_ emissions, showing the need for simpler and more affordable methods to measure individual animal emissions. Additionally, larger sample sizes, multitrait approaches, and indicator traits can improve the accuracy of GEBVs (Pickering et al., 2015; Manzanilla-Pech et al., 2022). Currently, the genetic architecture of CH_4_ phenotypes from GWAS is not extensively reported in the literature (Manzanilla-Pech et al., 2016; Manzanilla-Pech et al., 2022), and agreement is needed on which CH_4_ trait (production, yield, intensity, or other metrics) to use for genomic studies (Manzanilla-Pech et al., 2022). Once implemented, genomic selection provides an efficient approach to selection by determining the genotype early in life rather than direct measurement of CH_4_ emissions as the animal matures (Hayes et al., 2016; Pickering et al., 2015; Lassen and Difford, 2020). As CH_4_ measurement technologies improve and data on genotypes and phenotypes are acquired, CH_4_ emission reduction has strong potential for integration into breeding programs throughout the world.

## POTENTIAL TO COMBINE ENTERIC CH_4_ MITIGATION INTERVENTIONS

Overall, selective breeding could unlock the potential for lower CH_4_-emitting ruminants, while dietary reformulation and feed additives capitalize on their mitigation potential. In addition, the combination of interventions with different modes of action has the potential for additive enteric CH_4_ mitigation without compromising ruminant health. For example, it is proposed that increased ruminal H_2_ from 3-NOP could be utilized by interventions that create an alternative H_2_ pathway. Schilde et al. (2021) investigated the combined and separate effects of different levels of 3-NOP and concentrate inclusion in dairy cattle diets. The authors found the highest reductions in CH_4_ yield and intensity (approximately 33%) from the combination of 0.051 g 3-NOP/kg DM and 300–550 g concentrate/kg DM, with concentrate inclusion rate changing between pre- and post-calving diets (Schilde et al., 2021). Also, a combination of 3-NOP (0.200 g/kg DM) and lipids from canola oil (50 g/kg DM) fed to beef cattle resulted in approximately 51% reduction in CH_4_ yield, with the reduction increased incrementally from 3-NOP or lipids supplemented separately (Zhang et al., 2021). Lipids and nitrate have also been shown to additively (Guyader et al., 2015) and synergistically (Villar et al., 2020) reduce enteric CH_4_. However, the combination of lipids and nitrates also has the potential to decrease milk production (Guyader et al., 2016) and DM digestibility (Villar et al., 2020).

Studying potential combinations also elucidates interventions that interfere with each other, limiting or preventing additive CH_4_ reductions, such as tannin and lipid binding (Williams et al., 2020). Also, the addition of EO (0.150 g/kg DM) to a nitrate (17.9 g/kg DM) supplemented beef cattle diet did not further reduce enteric CH_4_, partially explained by the increase in CH_4_ from the chosen EO (Alemu et al., 2019). The authors also report no interaction effect between the EO and nitrate, showing potential for additive reductions with EO that work effectively to reduce CH_4_. Although grazing a combination of tanniferous legumes and alfalfa compared to a monoculture system resulted in no difference in enteric CH_4_ metrics, a greater ADG from the combined legumes would reduce time to slaughter and overall CH_4_ production from each animal (Lagrange et al., 2020).

In selective breeding, CH_4_ yield could be reduced by up to 45% from the combination of direct and indirect selection (Pickering et al., 2015), and the timeframe from this reduction will depend on impacts on production traits and the ability to phenotype and genotype a large reference population. Prior to adoption, combinations that are repeatedly shown to reduce CH_4_ emissions should be analyzed in an LCA to determine net environmental impact. Furthermore, most of the literature reports combinations for cattle, and more research is needed on small ruminants.

## FUTURE DIRECTIONS

**Figure 2.**
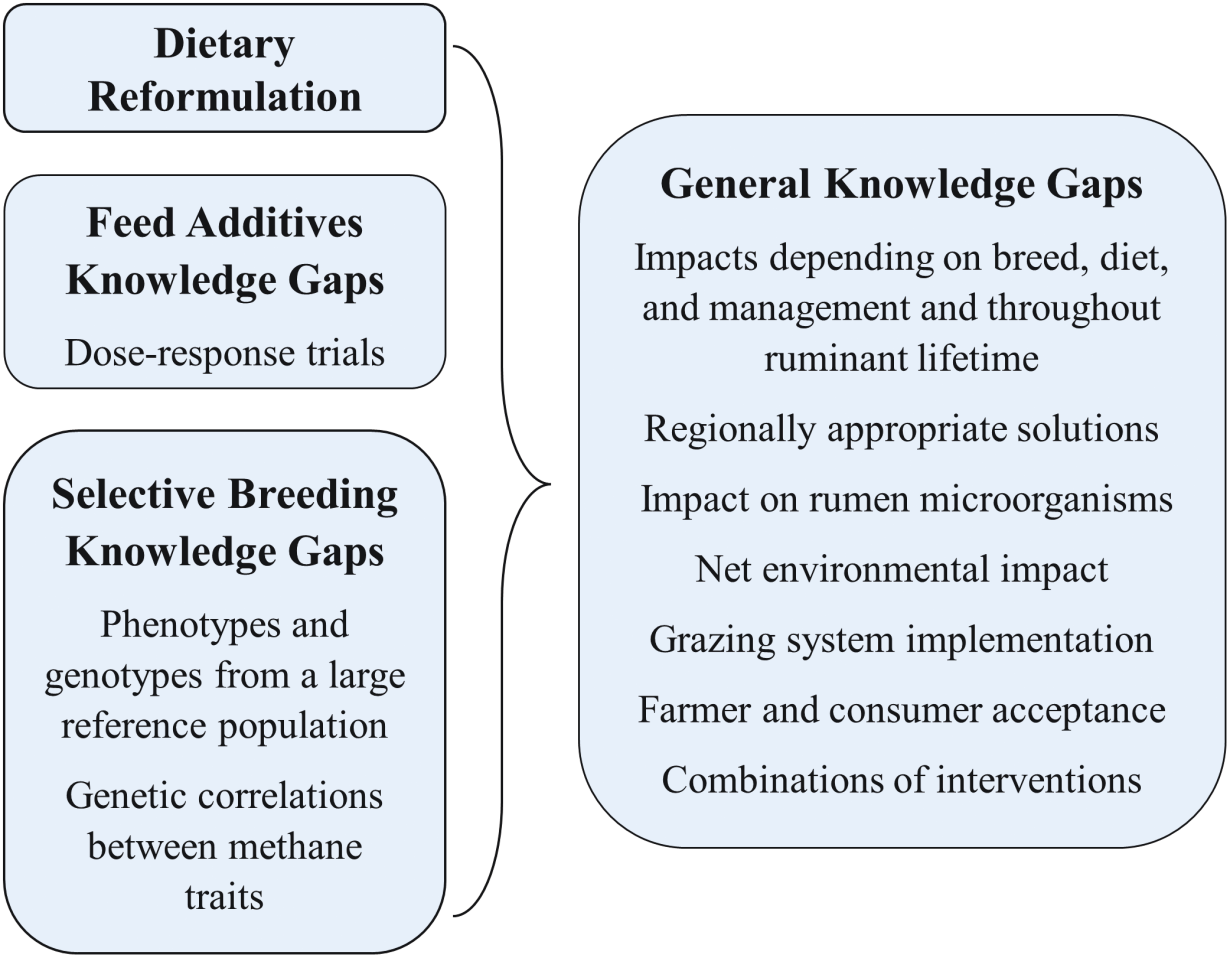
Current knowledge gaps of enteric methane mitigation interventions.

Dietary reformulation and feed additives allow for immediate reductions in enteric CH_4_, while selective breeding provides the advantage of long-term effects. The potential for reduction from these emerging interventions is met with the need for future research. Figure 2 depicts knowledge gaps from enteric CH_4_ mitigation interventions from the present review, including the need for regionally based solutions that best suit the animal’s breed, diet, and management (Dillon et al., 2021). Regionally appropriate interventions also depend on access to resources, which differs between low-, mid-, and high-income countries (Tricarico et al., 2020).

For feed additives, dose–response curves under a variety of dietary conditions can indicate appropriate inclusion rates. Future studies should also characterize the microorganisms in the rumen to further explain the mode of action and potential rumen adaptation (Newbold and Morales, 2020; Pitta et al., 2021). Furthermore, genomic selection requires a large reference population to be genotyped and phenotyped. Genetic correlations between CH_4_ and economically relevant traits should be better defined, as well as the consequences for these traits when CH_4_ is incorporated into the breeding goal (Beauchemin et al., 2020; Manzanilla-Pech et al., 2022).

For all interventions, future studies should investigate the impacts on CH_4_ reduction and health throughout the ruminant’s lifetime. Also, the net environmental impacts need quantification in various ruminant types, life stages, and diet compositions. Regionally based LCAs elucidate the opportunities to optimize the production and use of an intervention based on locally available resources and infrastructure (Nilsson and Martin, 2022). Additionally, modeling and communicating the GHG emissions associated with the research and widespread use of an intervention are crucial to define consequences on upstream and downstream emissions. Another research priority is the implementation of interventions in grazing systems and the resulting impacts on carbon sequestration, which is a growing area of opportunity for quantifying GHG reductions from livestock production (Dillon et al., 2021; Eugène et al., 2021).

Lastly, more research is needed on consumer and farmer acceptance of each CH_4_ mitigation intervention. Consumer acceptance of an intervention depends on effective communication on its safety and purpose. Farmer acceptance will be influenced by the economic implications of the intervention (including carbon credits and offset funding), as well as the impact on the management, cattle welfare, and goals of the farm.
